# The effects of *Saccharomyces cerevisiae* supplementation on intake, nutrient digestibility, and rumen fluid pH in Awassi female lambs

**DOI:** 10.14202/vetworld.2018.1015-1020

**Published:** 2018-07-30

**Authors:** Belal S. Obeidat, Kamel Z. Mahmoud, Mohammad D. Obeidat, Mysaa Ata, Rami T. Kridli, Serhan G. Haddad, Hosam H. Titi, Khaleel I. Jawasreh, Hosam J. Altamimi, Hadil S. Subih, Safaa M. Hatamleh, Majdi A. Abu Ishmais, Ruba Abu Affan

**Affiliations:** 1 Department of Animal Production, Faculty of Agriculture, Jordan University of Science and Technology, Irbid 22110, Jordan; 2 Department of Animal Production and Protection, Faculty of Agriculture, Jerash University, Jerash 26150, Jordan; 3 Department of Animal Production, Faculty of Agriculture, University of Jordan, Amman 11942, Jordan; 4 Department of Nutrition and Food Technology, Faculty of Agriculture, Jordan University of Science and Technology, Irbid 22110, Jordan

**Keywords:** Awassi female lamb, intake, nutrients digestibility, yeast supplementation

## Abstract

**Aim:**

The aim of this study was to evaluate the effect of feeding low (LO)- or high (HI)-fiber diets supplemented with *Saccharomyces cerevisiae* (SC) on nutrient intake, digestibility, nitrogen balance, rumen fluid pH, and serum concentrations of glucose and urea nitrogen in Awassi female lambs in a 2×2 factorial arrangement of treatments.

**Materials and Methods:**

Experimental diets were as follows: (1) LO-fiber diet with no SC supplementation (−LO), (2) LO-fiber diet supplemented with SC (+LO), (3) HI-fiber diet with no SC supplementation (−HI), or (4) HI-fiber diet supplemented with SC (+HI). Eight female lambs were used in a replicated 4×4 Latin square design with 15-day experimental periods (10-day adaptation period and 5-day collection period).

**Results:**

A fiber×SC interaction (p≤0.05) was detected for dry matter (DM) and crude protein (CP) intake among diets showing greater DM and CP intake for +LO diet compared to +HI group supplemented with SC, whereas −LO and −HI were intermediate. A fiber×SC interaction (p=0.05) was also detected for the neutral detergent fiber (NDF) intake among diets. Intake of NDF was greater for the –HI diet compared with +LO and –LO diets. Similarly, NDF intake was greater for +HI diet than –LO diet. A tendency (p=0.07) for a fiber×SC interaction was detected for acid detergent fiber (ADF) intake among diets as well. ADF intake tended to be greater for HI-fiber diets. No difference was observed in the rumen fluid pH for lambs fed with the different diets. No fiber×SC interactions were detected for the digestibility of DM, CP, NDF, and ADF among dietary treatments. Digestibility of DM was greater (72.9 g/100 g vs. 67.1 g/100 g; p=0.0002) for LO versus HI fiber. However, NDF and ADF digestibilities were greater (60.8 and 61.9 g/100 g vs. 55.8 and 52.7 g/100 g for NDF and ADF digestibility, respectively; p≤0.01) for the HI-fiber than the LO-fiber diets.

**Conclusion:**

Results obtained in the current study indicate that SC supplementation has a minimal effect on the performance of Awassi female lambs fed with varying fiber levels.

## Introduction

Nutritional programs for ruminant animals focus on providing the precise level of nutrients at the proper time, to obtain optimal productivity and profitability [1]. Level and type of forages are among the most important factors that determine the effect of feed additives, such as yeast, on animal performance [2]. The most widely used additives in animal nutrition over the past decade are probiotics. Probiotics are live microorganisms that can provide a health benefit for the host animals and improve forage digestibility as well [3]. *Saccharomyces cerevisiae* (SC) has been found to improve ruminal fermentation [4], enhance microbial growth, and improve the stability of rumen fermentation. Moreover, supplementing yeast provides some nutrients during digestion which impact the rumen microbial population and function.

Many studies showed beneficial effects of yeast on the ruminal environment, the number of ruminal microbes, and microbial activity [5-7]. The number of ruminal microbes and cellulolytic bacteria increased with dietary yeast supplementation [8], due to the increase in the population of beneficial microorganisms. Adding yeast to dairy cattle diets was reported to enhance the overall performance through improving the efficiency of nutrient utilization [9].

Supplementing yeast with high (HI)- and low (LO)-fiber diets in sheep can test the hypothesis that the level of the fiber in the diet can impact the efficacy of yeast on performance. Therefore, this study aimed to evaluate the influence of SC supplementation on diets containing LO versus HI neutral detergent fiber (NDF) on nutrient intakes and digestibility, nitrogen balance, and rumen fluid pH in Awassi female lambs.

## Materials and Methods

### Ethical approval

The study was conducted at the Agricultural Research and Training Unit at Jordan University of Science and Technology (JUST). All procedures used in the study were approved by the JUST Institutional Animal Care and Use Committee (16/3/3/222).

### Experiment procedures

Eight Awassi female lambs (6 months old) were used in 2×2 factorial arrangements to evaluate the effect of yeast (Levucell^®^SC, Lallemand, France; 3×10^9^/g) supplementation on the criteria listed above. Animals were housed in metabolic crates (1.05 m×0.80 m) and offered diets containing either LO or HI level of fiber (HI). Diets were as follows: (1) LO-fiber diet with no SC supplementation (−LO), (2) LO-fiber diet supplemented with SC (+LO), (3) HI-fiber diet with no SC supplementation (−HI), or (4) HI-fiber diet supplemented with SC (+HI). To ensure complete consumption, SC (0.25 g/head/day+10 g ground barley) was mixed with 50 g of the assigned diet throughout the experimental period. Diets were formulated to have similar crude protein (CP) contents (analyzed; 140 g/kg CP; dry matter [DM] basis). Diets were offered *ad libitum* (at 8:00 h) with free access to water throughout the study. The study was designed with four experimental periods; each period lasted for 15 days (10 days were used as adaptation to the metabolic crates and the experimental diets followed by 5 days for data collection). Offered and refused feeds were recorded from days 10 to 15 of each experimental period to evaluate intake.

Total fecal output was collected, weighed, and recorded, and then, a sample of 10 g/100 g of each was kept for subsequent chemical analysis during the period from days 10 to 15. During the collection period, urine was collected daily in buckets (containing 50 ml 1 N HCl), weighed, and recorded, and 5 g/100 g of each was kept for nitrogen analysis. On day 15 of each experimental period, 5 ml of rumen fluid was collected from the ventral sac using a syringe fitted with a G14 needle at 0, 2, and 4 h after feeding (at 12: 00 h) to evaluate rumen fluid pH.

Following the Association of Official Analytical Chemists (AOAC) [10] procedures, diets and fecal samples were analyzed for DM, CP, NDF, and acid detergent fiber (ADF). Ingredient and chemical composition of the LO- and HI-fiber diets are presented in Table-1. At the end of each period, 10 ml of blood samples were collected from the jugular vein to evaluate blood metabolites (glucose and urea nitrogen).

**Table-1:** Ingredients and chemical composition of the LO- and HI-fiber diets fed to Awassi female lambs.

Ingredient, % DM	LO	HI
Barley grain	53	26
Soybean meal (440 g/kg CP; solvent)	11	17
Alfalfa hay	14	12
Wheat straw	20	43
Salt	1	1
Limestone	1	1
Vitamin/mineral premi×^1^	+	+

**Analyzed chemical composition, (%)**		

DM	91.6	92.7
CP	14.1	13.8
NDF	37.6	52.0
ADF	17.8	29.1
ME, Mcal/kg	2.3	2.1

1 One kg was added for each 1000 kg mixed. Composition per kg contained (Vitamin A, 600,000 IU; Vitamin D_3_, 200,000 IU; Vitamin E, 75 mg, Vitamin K3, 200 mg; Vitamin B1, 100 mg; Vitamin B5, 500 mg; lysine 0.5%; DL-methionine, 0.15%; manganese oxide, 4000 mg; ferrous sulfate, 15,000 mg; zinc oxide, 7000; magnesium oxide, 4000 mg; potassium iodide, 80 mg; sodium selenite, 150 mg; copper sulfate, 100 mg; cobalt phosphate, 50 mg, dicalcium phosphate, 10,000 mg. DM=Dry matter, CP=Crude protein, NDF=Neutral detergent fiber, ADF=Acid detergent fiber, ME=Metabolizable energy, LO=Low, HI=High

### Laboratory procedures

At the end of the experiment, feed and fecal samples were dried at 55°C in a forced air oven to constant weight and ground to pass a 1 mm sieve (Brabender, Duisburg, Germany). These samples were analyzed for DM (100°C in an air-forced oven for 24 h), and nitrogen (Kjeldahl procedure; ^#^976.06) using AOAC procedures [10]. NDF and ADF analyses were performed using the Ankom2000 fiber analyzer apparatus (Ankom Technology Corporation, Macedon, NY, USA). The NDF analysis used sodium sulfite in the neutral detergent solution and a heat stable alpha-amylase. Both NDF and ADF are expressed with residual ash.

Blood samples were centrifuged (at 3000 rpm, for 15 min) after 1 h of collection. Serum was immediately harvested and stored at −20°C until the day of analysis. Serum concentrations of glucose and urea nitrogen were analyzed using commercial kits (BioSystems, S. A. Costa Brava, Barcelona, Spain) and according to the manufacturer’s specifications. Absorbance was measured using a spectrophotometer (JENWAY 6105 ultraviolet/visible, Model 6105, Jenway Ltd., Felsted, Dunmow ESSEX CM6 3LB, UK).

### Statistical analysis

This experiment was designed to test the effect of fiber levels during the presence or absence of yeast (2×2 factorial treatments) by running two 4×4 Latin squares. Four animals were used in each square and sampled over four experimental periods (15 days for each period). Data analyses were conducted on measurements collected from pooled samples during day 11–15 of the experimental periods. Experimental unit measurements were analyzed using the mixed model procedure of SAS (PROC MIXED; SAS Inc., Cary, NC, 2000) to account for the effects of square, animal within square, period, and treatment (2×2 factorial; fiber levels vs. presence or absence of SC). Treatments were considered fixed effect; square, animal within square, and period were considered as random effects. Data are presented as least square means±standard error of the mean. Statistical significance was considered at p≤0.05.

## Results

The effect of SC supplementation on nutrient intakes and rumen fluid pH is presented in Table-2. The results clearly show that sheep fed with LO-fiber diets consumed more (p≤0.05) DM and CP; however, this increase was dependent on the presence or absence of SC. Adding SC to LO-fiber rations increased DM and CP intakes compared to their HI-fiber diet counterparts due to fiber×SC interaction (p≤0.05). A fiber×SC interaction (p=0.05) was also detected for NDF intake among diets. Intake of NDF was greater for the −HI diet compared with +LO and −LO diets. Similarly, NDF intake was greater for the +HI than the −LO diet. There was a tendency (p≤0.07) for the fiber×SC interaction to affect ADF intake. ADF intake was greater in lambs fed with HI-fiber diets. No difference was observed for the rumen fluid pH for lambs fed with the different diets.

**Table-2:** Effect of SC supplementation in Awassi female lambs fed with LO- and HI-fiber diets on nutrient intakes (g/kg DM) and rumen fluid pH.

Item	Fiber^1^				SEM	p-value		

	LO		HI					
		
	+LO	−LO	+HI	−HI		Fiber	SC	Fiber×SC

Intake, g/kg								
DM	1390^b^	1265^ab^	1143^a^	1281^ab^	78.9	0.08	0.91	0.048
CP	196^b^	178^ab^	158^a^	177^ab^	11.0	0.03	0.93	0.05
NDF	522^bc^	475^c^	595^ab^	666^a^	36.6	0.001	0.67	0.05
ADF	247^a^	225^a^	332^b^	372^b^	19.7	<0.01	0.58	0.07
Rumen fluid pH	6.1	6.0	6.4	6.4	0.14	0.25	0.58	0.21

1 Diets were as follows: (1) LO-fiber diet with no SC supplementation (−LO), (2) LO-fiber diet supplemented with SC (+LO), (3) HI-fiber diet with no SC supplementation (−HI), or (4) HI-fiber diet supplemented with SC (+HI). DM=Dry matter, CP=Crude protein, NDF=Neutral detergent fiber, ADF=Acid detergent fiber, LO=Low, HI=High, SC=*Saccharomyces cerevisiae*, SEM=Standard error of the mean

The digestibility of DM, CP, NDF, and ADF is presented in Table-3. Female lambs consumed different diets had a similar CP digestibility; however, fiber content significantly altered DM, NDF, and ADF digestibilities. DM digestibility increased (p<0.01) when sheep consumed LO-fiber diets compared to their counterparts on HI-fiber diets. To the contrary, HI-fiber diet was associated with higher (p<0.05) digestibility of NDF and ADF. SC supplementation did not change the digestibility of the discussed variables.

**Table-3:** Effect of SC supplementation in Awassi female lambs fed with LO- and HI-fiber diets on nutrient digestibility (%).

Item	Fiber^1^				SEM	p-value		

	LO		HI					
		
	+LO	−LO	+HI	−HI		Fiber	SC	Fiber×SC
DM	72.7	73.1	66.0	68.2	1.22	<0.001	0.27	0.41
CP	72.8	71.9	70.2	74.5	1.90	0.99	0.39	0.20
NDF	55.1	56.4	59.8	61.9	1.93	0.011	0.33	0.84
ADF	51.0	54.4	60.9	63.0	2.03	<0.001	0.13	0.70

1 Diets were as follows: (1) LO-fiber diet with no SC supplementation (−LO), (2) LO-fiber diet supplemented with SC (+LO), (3) HI-fiber diet with no SC supplementation (−HI), or (4) HI-fiber diet supplemented with SC (+HI). DM=Dry matter, CP=Crude protein, NDF=Neutral detergent fiber, ADF=Acid detergent fiber, LO=Low, HI=High, SC=*Saccharomyces cerevisiae*, SEM=Standard error of the mean

The effects of SC supplementation in LO- or HI-fiber diets on nitrogen balance are shown in Table-4. Female lambs fed with LO-fiber diets maintained higher (p<0.05) nitrogen intake and excreted more (p<0.05) as well; however, these groups showed higher (p<0.05) nitrogen retention which was associated with lower (p<0.05) nitrogen losses through urine. There was a clear fiber×SC interaction (p<0.05) for the nitrogen intake and an interaction tendency for the nitrogen retention variable. The SC supplementation increased nitrogen intake in groups fed with the LO-fiber diet and relatively increased its body retention. No fiber×SC interaction was detected for nitrogen excreted in feces or urine.

**Table-4:** Effect of SC supplementation in Awassi female lambs fed with LO- and HI-fiber diets on nitrogen balance (g/d).

Item	Fiber^1^				SEM	p-value		

	LO		HI					
		
	+LO	−LO	+HI	−HI		Fiber	SC	Fiber×SC
N intake	31.4^a^	28.5^ab^	25.3^b^	28.3^ab^	1.76	0.03	0.94	0.046
N in feces	8.5	8.1	7.3	7.3	0.52	0.01	0.61	0.47
N in urine	6.6	6.4	7.8	8.1	0.38	0.002	0.89	0.53
N retained	16.3^a^	14.1^a^	10.2^b^	12.9^ab^	1.39	0.01	0.87	0.07

1 Diets were as follows: (1) LO-fiber diet with no SC supplementation (−LO), (2) LO-fiber diet supplemented with SC (+LO), (3) HI-fiber diet with no SC supplementation (-−HI), or (4) HI-fiber diet supplemented with SC (+HI), LO=Low, HI=High, SC=*Saccharomyces cerevisiae*, SEM=Standard error of the mean

No fiber×SC supplementation interaction was detected for serum concentrations of glucose and urea nitrogen among diets (Table-5). Similarly, serum concentrations of glucose and urea nitrogen were not affected by the level of fiber or SC supplementation.

**Table-5:** Effect of SC supplementation in Awassi female lambs fed with LO- and HI-fiber diets on serum concentrations (mg/dL) of glucose and urea N.

Item	Fiber^1^				SEM	p-value		

	LO		HI					
		
	+LO	−LO	+HI	−HI		Fiber	SC	Fiber×SC
Glucose	56.4	54.4	50.3	52.8	3.46	0.17	0.93	0.42
Urea N	19.6	18.1	19.0	18.3	1.23	0.89	0.37	0.75

1 Diets were as follows: (1) LO-fiber diet with no SC supplementation (−LO), (2) LO-fiber diet supplemented with SC (+LO), (3) HI-fiber diet with no SC supplementation (−HI), or (4) HI-fiber diet supplemented with SC (+HI), LO=Low, HI=High, SC=*Saccharomyces cerevisiae*, SEM=Standard error of the mean

## Discussion

The use of yeast as an additive for growing lambs is to optimize the use of dietary fiber, resulting in improved growth. Moreover, yeast may affect ruminal pH by reducing the activity of lactic acid producing bacteria and thus increasing its nutrient availability [11]. In the current study, DM and CP intakes were greater for animals fed with LO-fiber diets supplemented with SC (+LO). This indicated that the level of fiber has a detrimental effect on DM intake, consistent with results of previous studies [12,13]. Hassan and Mohammed [14] found that DM intake was not affected by yeast addition to different roughage to concentrate ratios while CP intake increased when diets contained less fiber. Consistent with these results, intake did not change by adding SC to diets with different roughage to concentrate [15] and when added to concentrate diets [16] in male lambs. On the contrary, Hansen *et al*. [17] reported an increase in DM intake when adding yeast culture to diets fed to buffaloes.

Moreover, an interaction between SC supplementation and daily feed intake of ADF and NDF was previously reported [15]. CP intake increased with HI concentrates supplemented with SC, whereas ADF and NDF increased with HI roughage supplemented with SC. Those findings might be due to the stabilization of ruminal fermentation and activity due to SC supplementation, which might have enhanced the intake of CP [18]. Ghasemi *et al*. [19] observed that SC supplementation was linked with increased NDF intake. The increase in ADF intake could be due to SC addition and fiber content, which can be attributed to the improvement of the digestibility of this nutrient (Table-3). In general, any increase in the rate of digestion should enhance the rate of passage that increases animal intake of nutrients [20].

Contrary to our finding, Hasunuma *et al*. [21] found that DM intake was not affected by yeast supplementation to dairy cattle. Furthermore, Whitley *et al*. [22] reported that DM intake was not impacted by yeast supplementation due to the limited involvement of yeast with ruminal microbes, which is required for improving ruminal fermentation and increasing intake.

Results reported in the present study revealed no fiber×SC interactions with respect to digestibility of DM, CP, NDF, and ADF. Digestibility of nutrients is enhanced by the type of fiber used. Bueno *et al*. [23] reported similar results to ours. They found that the interaction between adding yeast and diets with different levels of fiber to concentrate ratios was not statistically significant, which indicates that active yeast supplementation was effective on the two diets independent of their fiber to concentrate ratio. Active yeast can improve the ruminal environment, mainly by keeping pH values around neutrality and avoiding drastic pH drop in diets with LO-fiber proportions [24]. Other researchers suggested that yeast culture inclusion has an important role in nutrient digestion for animals maintained on HI-forage diets [25]. Intake and digestion of feeds are dependent on the initial rate of fiber digestion; early stimulus of ruminal action would positively enhance feed consumption and might improve animal performance [26].

Digestibility of CP was not affected in lambs fed with diets supplemented with SC or by the type of fiber used, while NDF and ADF digestibilities improved in lambs fed with HI-fiber diets. Wiedmeier *et al*. [27] found higher CP digestibility values in cattle fed with yeast-supplemented rations. This supplementation might stimulate proteolytic bacteria that significantly increase CP digestibility. In addition, Wohlt *et al*. [28] reported that yeast supplementation improves CP digestibility and, as a result, DM intake in cows. Other researchers reported that some yeast cultures impacted the number of cellulolytic bacteria in the rumen and thus increased cellulose degradation. They also suggested that SC supplementation stimulates the rate, rather than the extent, of fiber digestion in the rumen by its microorganisms [4].

A fiber×SC supplementation interaction was detected for the nitrogen intake among diets, showing that nitrogen intake was improved with the different fiber levels. These results contradict those of Bueno *et al*. [23] who found that nitrogen balance was not modified by active yeast supplementation regardless of the levels of fed fiber. Fecal nitrogen excretion was improved, and urinary nitrogen excretion was reduced for female lambs fed with the LO diet compared to female lambs fed with the HI diet. Gad *et al*. [29] reported that nitrogen balance improves with lower than HI dietary fiber and attributed this finding to the improvement of CP digestibility. Furthermore, Cole *et al*. [30] reported that lambs raised on rations supplemented with yeast had greater nitrogen -balance than controls. One probable explanation for the increase in the nitrogen balance could be related to the improvement in the production of microbes and, as a result, increased production of microbial protein. In addition, the improvement in nitrogen balance could be related to the increase in the utilization of ammonia [31] and/or due to the availability of fermentable carbohydrates in the rumen.

The addition of SC to female lamb diets containing different fiber levels did not affect serum concentrations of glucose and urea nitrogen. Our results are in agreement with the results obtained by Khormizi *et al*. [32] who indicated that plasma content of glucose and urea nitrogen was not affected by supplementation with yeast. Raghab *et al*. [33], on the other hand, recorded that dietary supplementation of yeast resulted in a significant increase in serum glucose concentration in calves. The lack of effect of SC supplementation on these parameters in this experiment might be due to the inability of yeast to influence protein and fibers degradation.

## Conclusion

Supplementing SC has a minimal effect on the performance (intake, digestibility, and nitrogen retention as well as blood metabolites) of Awassi female lambs fed with two levels of fiber. The relationships between SC supplementation and fiber levels are worth further investigation to determine the situations where supplemental SC is expected to improve animal performance.

## Authors’ Contributions

BSO: Designing, supervising, and writing the manuscript, KZM: Helping in statistical analysis and writing the manuscript, MDO: Drafting the manuscript, MA: Drafting the manuscript, RTK: Helping in technical writing, SGH: Drafting the manuscript, HHT: Drafting the manuscript, KIJ: Drafting the manuscript, HJA: Drafting The manuscript, HSS: Drafting the manuscript, SMH: Field work, MAAI: Doing the laboratory analyses. RAA: Field work. All authors read and approved the final manuscript.
